# Keratin 6 is not essential for mammary gland development

**DOI:** 10.1186/bcr1504

**Published:** 2006-06-21

**Authors:** Sandra L Grimm, Wen Bu, Mary Ann Longley, Dennis R Roop, Yi Li, Jeffrey M Rosen

**Affiliations:** 1 Department of Molecular and Cellular Biology, Baylor College of Medicine, One Baylor Plaza, Houston, TX 77030, USA; 2 Breast Center, Baylor College of Medicine, One Baylor Plaza, Houston, TX 77030, USA

## Abstract

**Introduction:**

Keratin 6 (K6) has previously been identified as a marker of early mammary gland development and has also been proposed to be a marker of mammary gland progenitor cells. However, the function of K6 in the mammary gland was not known, so we examined the expression pattern of the protein during both embryonic and postnatal mammary development, as well as the mammary gland phenotype of mice that were null for both K6a and K6b isoforms.

**Method:**

Immunostaining was performed to determine the expression pattern of K6a throughout mammary gland development, from the embryonic mammary bud to lactation. Double immunofluorescence was used to co-localize K6 with known markers of mammary gland development. Wild-type and K6ab-null mammary tissues were transplanted into the cleared fat pads of nude mice and the outgrowths were analyzed for morphology by whole-mount staining and for markers of mammary epithelium by immunostaining. Finally, progesterone receptor (PR) and bromodeoxyuridine co-localization was quantified by double immunofluorescence in wild-type and K6ab-null mammary outgrowths.

**Results:**

Here we report that K6 is expressed earlier than described previously, by embryonic day 16.5. K6a is the predominant isoform expressed in the mammary gland, localized in the body cells and luminal epithelial cells but not in the cap cells or myoepithelial cells. Co-localization studies showed that most K6a-positive cells express steroid receptors but do not proliferate. When both the K6a and K6b genes are deleted, mammary gland development appears normal, with similar expression of most molecular markers examined in both the pubertal gland and the mature gland. Loss of K6a and K6b, however, leads to an increase in the number of steroid-receptor-positive cells, and increased co-localization of steroid receptor expression and proliferation was observed.

**Conclusion:**

Although K6a was not essential for mammary gland development, loss of both K6a and K6b resulted in an increase in PR-positive mammary epithelial cells and decreased proliferation after exposure to steroid hormones. There was also increased co-localization of PR and bromodeoxyuridine, suggesting alterations in patterning events important for normal lobuloalveolar development.

## Introduction

The mammary gland is unique in that its development primarily occurs postnatally. However, the tissue is initially formed during embryonic development (reviewed in [1]). A milk line first appears at about embryonic day 10.5 (E10.5). At E11.5, five pairs of placodes have formed at specific positions along the milk line, and by E12.5 mammary buds invaginate from the ectoderm, surrounded by a specialized mammary mesenchyme. These mammary anlagen begin to form a lumen by E16.5 and sprout into the underlying fat pad. Branching morphogenesis then occurs, to give rise to a rudimentary ductal tree in the newborn pups.

Any piece of the mammary gland, from the embryonic mammary bud to the differentiated gland, can be transplanted into a cleared fat pat to generate another ductal structure containing all the epithelial cell types that make up the mammary gland, supporting the idea that progenitor cells are dispersed throughout the tissue [2]. Although lineage markers have been identified in the hematopoietic system and epidermis, a clear picture of mammary lineage markers is still evolving [3-5]. However, a handful of putative markers have been identified, including keratin 6 (K6) [6,7].

Cytokeratins, members of the intermediate filament superfamily, are the main structural components of most epithelial cells. There are more than 55 keratins, consisting of type I (K9 to K20) and type II (K1 to K8) filaments that partner to form coiled-coil heterodimers [8]. Although multiple genes encoding K6 isoforms exist in both human and mouse, the mouse genes seem to have evolved after the species diverged [9]. The mouse isoforms, K6a and K6b, are organized in tandem on chromosome 15 and although their coding sequences show 95% identity, the two genes are differentially regulated at the transcriptional level [10-12]. Germline deletion of both K6a and K6b genes led to the discovery of a third murine isoform, K6hf, expressed mainly in hair follicles [12]. Expression of K6 in the skin is associated with hyperproliferative disorders and in response to stressful stimuli, such as wounding [13]. After an injury, K6 is expressed at the wound site, where its expression is associated with activated keratinocytes [14]. However, the function of K6 in mammary gland development is not known.

K6 is expressed in terminal end buds (TEBs) of the developing mammary gland [15,16]. Ductal elongation is a highly proliferative phase of mammary gland development, but K6 expression is restricted to the body cells of the TEB and not the proliferative cap cell layer surrounding the tip of the TEB. However, expression of K6 is rare in the mature mammary gland [7,16]. Additionally, K6 is misexpressed in mature mammary glands from mice that were null for CCAAT-enhancer binding protein-β (C/EBPβ), coinciding with an arrested state of differentiation and a block in cell fate [6].

The function of K6 in the mammary gland is not known, so we examined the expression pattern throughout mammary development, as well as the mammary gland phenotype of mice that are null for both K6a and K6b isoforms (K6ab-null). Here we report that K6 is expressed earlier than described previously, by E16.5. K6a is the predominant isoform expressed in the mammary gland, localized in the body cells and luminal epithelial cells but not in the cap cells or myoepithelial cells. Co-localization studies showed that most K6a-positive cells express steroid receptors but do not proliferate. When both the K6a and K6b genes are deleted, mammary gland development appears normal, with similar expression of most molecular markers examined in both the pubertal gland and the mature gland. However, an increase in the number of steroid-receptor-positive cells and increased co-localization of steroid receptor expression with proliferation were observed.

## Materials and methods

### Animals and tissue isolation

Animal care and procedures were approved by the Institutional Animal Care and Use Committee of Baylor College of Medicine and were in accordance with the procedures detailed in the Guide for Care and Use of Laboratory Animals (NIH publication 85–23). Mice with targeted germline deletion of K6a and K6b have been described previously [12]. Mammary glands were removed from intact 6-week-old wild-type (WT) and K6ab-null mice after an intraperitoneal injection with 5-bromo-2'-deoxyuridine (BrdU; 0.03 mg/g body weight; Sigma, St Louis, MO, USA) 2 hours before being killed. Mammary glands were also isolated from C57Bl/6 mice at various stages of development (embryonic days 14 and 16.5, day 5 pups, 6 and 12 weeks, days 10, 15, 18 of pregnancy, days 2 and 9 of lactation and day 3 of involution). After fixation for 2 hours in cold 4% paraformaldehyde, the tissues were embedded in paraffin. Mammary glands and skin samples were additionally collected from 6-week-old FVB females that were injected intraperitoneally with BrdU 2 hours before being killed. These tissues were either fixed overnight in 10% normal-buffered formalin (NBF) and embedded in paraffin or frozen in OCT (Optimal Cutting Temperature) for cryosectioning. Paraffin-embedded mammary glands and skin samples were sectioned (5 to 7 μm) onto Probe-On Plus charged slides (Fisher Scientific, Pittsburgh, PA, USA). Frozen sections were cut at 5 μm thickness and fixed in acetone for 10 minutes.

### Mammary gland transplants

Mammary gland tissues from adult WT or K6ab-null mice were cut into small pieces (about 1 mm^3^) that were inserted into the cleared fat pads of 3-week-old athymic *nu*/*nu *mice [17]. All clearings were subjected to whole-mount staining to verify that all epithelium was removed from the fat pad. Primary transplants were allowed to grow out for at least 16 weeks so that the fat pad was completely filled with epithelium. These outgrowths were removed, cut into small pieces, and slowly frozen in RPMI-1640 medium containing 2% fetal bovine serum with 7% dimethylsulphoxide and used to perform secondary transplants. Outgrowths were taken at 4 and 10 weeks after transplantation. At 10 weeks after transplantation, the host mice were treated for 48 hours with a single interscapular subcutaneous injection of 17β-estradiol benzoate (1 μg) and progesterone (1 mg) in 100 μl of sesame oil (all from Sigma). At 2 hours before being killed, animals were injected with BrdU. After fixation for 2 hours in cold 4% paraformaldehyde, the glands were cut in half lengthways. One half was embedded in paraffin and the other was subjected to hematoxylin whole-mount staining. Whole-mount images were captured with an Olympus SZ40 dissecting microscope connected to a QCapture digital camera.

### Antibodies and immunostaining

Paraffin-embedded tissue sections were deparaffinized in xylene, then rehydrated through a graded ethanol series. Immunostaining was performed after microwave antigen retrieval (20 minutes) in 10 mM sodium citrate and blocking in 5% bovine serum albumin in phosphate-buffered saline containing 0.5% Tween 20. For immunohistochemistry, sections were incubated overnight with the following primary antibodies at room temperature: K6 rabbit polyclonal at a dilution of 1:5000 (Covance, Richmond, CA, USA), keratin 5 (K5) rabbit polyclonal at 1:5000 (Covance), keratin 8 (K8) rat monoclonal at 1:5000 (University of Iowa Developmental Studies Hybridoma Bank), estrogen receptor α (ERα) rabbit polyclonal at 1:5000 (Santa Cruz Biotechnology, Santa Cruz, CA, USA), and biotinylated BrdU mouse monoclonal at 1:50 (BD Biosciences, San Jose, CA, USA). Immunoperoxidase staining was detected using the appropriate biotinylated secondary antibodies and Vectastain Elite ABC and diaminobenzidine substrate kits in accordance with manufacturer's instructions (Vector Laboratories, Burlingame, CA, USA). For immunofluorescence, sections were incubated overnight with the following primary antibodies at room temperature: K6a rabbit polyclonal at 1:100 (Covance), K6b/hf guinea pig polyclonal at 1:100 [11], smooth muscle α-actin (SMA) mouse monoclonal at 1:100 (Dako, Carpinteria, CA, USA), K8 rat monoclonal at 1:100 (University of Iowa Developmental Studies Hybridoma Bank), ERα rabbit polyclonal at 1:1000 (Santa Cruz Biotechnology), fluorescein isothiocyanate-conjugated BrdU at 1:10 (BD Biosciences), and progesterone receptor (PR) rabbit polyclonal at 1:50 (Dako). Immunofluorescence staining was detected with the appropriate secondary antibodies conjugated with Texas Red, Alexa 568, or Alexa 488 (Molecular Probes, Eugene, OR, USA), and nuclei were counterstained with 4', 6-diamidino-2-phenylindole (DAPI; Vector Laboratories).

### Image capture, cell counting and statistical analysis

An Olympus BX40 light microscope connected to a MagnaFire digital camera was used to capture images from immunohistochemical staining. A Zeiss Axioskop2 Plus fluorescence microscope connected to an AxioCamMR digital camera was used to capture images from immunofluorescence staining with Axiovision Rel. 4.2 software. At least eight individual 40× fields per group were captured for counting. K6a-positive cells were scored and then the number of cells expressing ERα or BrdU in that subset was counted. Fluorescent images of PR and BrdU immunostaining were captured digitally with an Olympus BX50 microscope connected to a Hamamatsu C5810 charge-coupled device camera. At least eight individual 60× fields per sample were captured for counting. The number of positively stained mammary epithelial cell (MEC) nuclei was expressed as a percentage of the total number of luminal epithelial cells. Statistical significance was determined by Student's *t *test (two-sample assuming unequal variance).

## Results

**Figure 1:**
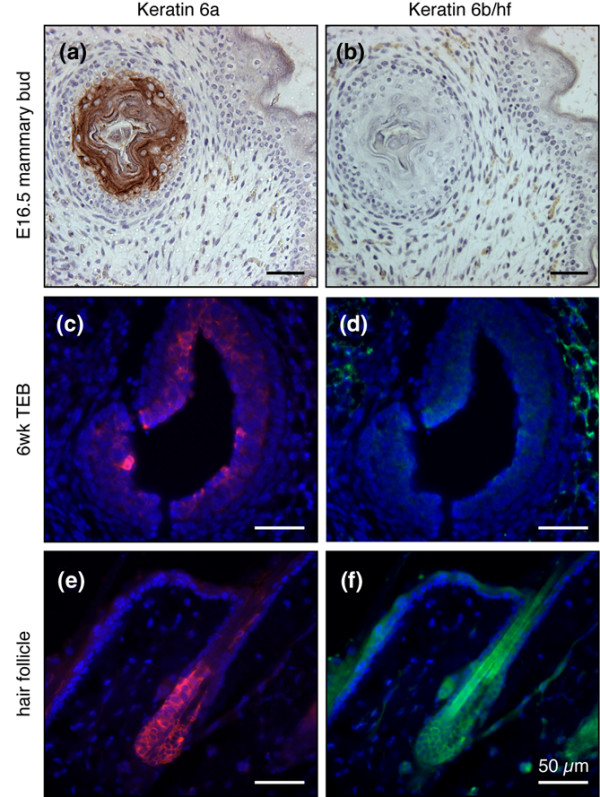
Keratin 6a (K6a) is the predominant isoform expressed in the mammary gland. Antibodies that recognize either the K6a or K6b/hf isoform were used to immunostain sections from either E16.5 embryonic mammary buds **(a,b)**, terminal end buds (TEBs) from 6-week FVB mouse mammary glands **(c,d) **or hair follicles **(e,f)**. K6a stained the mammary bud and body cells of the TEB as well as the hair follicle, but Kb6/hf was detected only in the hair follicle. Scale bar, 50 μm.

Three isoforms of mouse K6 exist, namely K6a, K6b and K6hf [12]. To determine which isoforms are expressed in the mammary gland, immunostaining was performed on tissue sections with the use of antibodies that specifically recognize either K6 or K6b/hf (Figure 1). An embryonic mammary bud from E16.5 stained positively for K6a (Figure 1a) but an adjacent section showed no immunoreactivity with the K6b/hf antibody (Figure 1b). Indirect immunofluorescence revealed K6a-positive body cells in a TEB from a 6-week mammary gland (Figure 1c), but no K6b/hf staining was detected (Figure 1d). A positive control is shown in Figure 1e,f, in which both antibodies stained the outer root sheath of a hair follicle from a 6-week skin sample. K6a is therefore the predominant isoform expressed in the mouse mammary gland.

**Figure 2:**
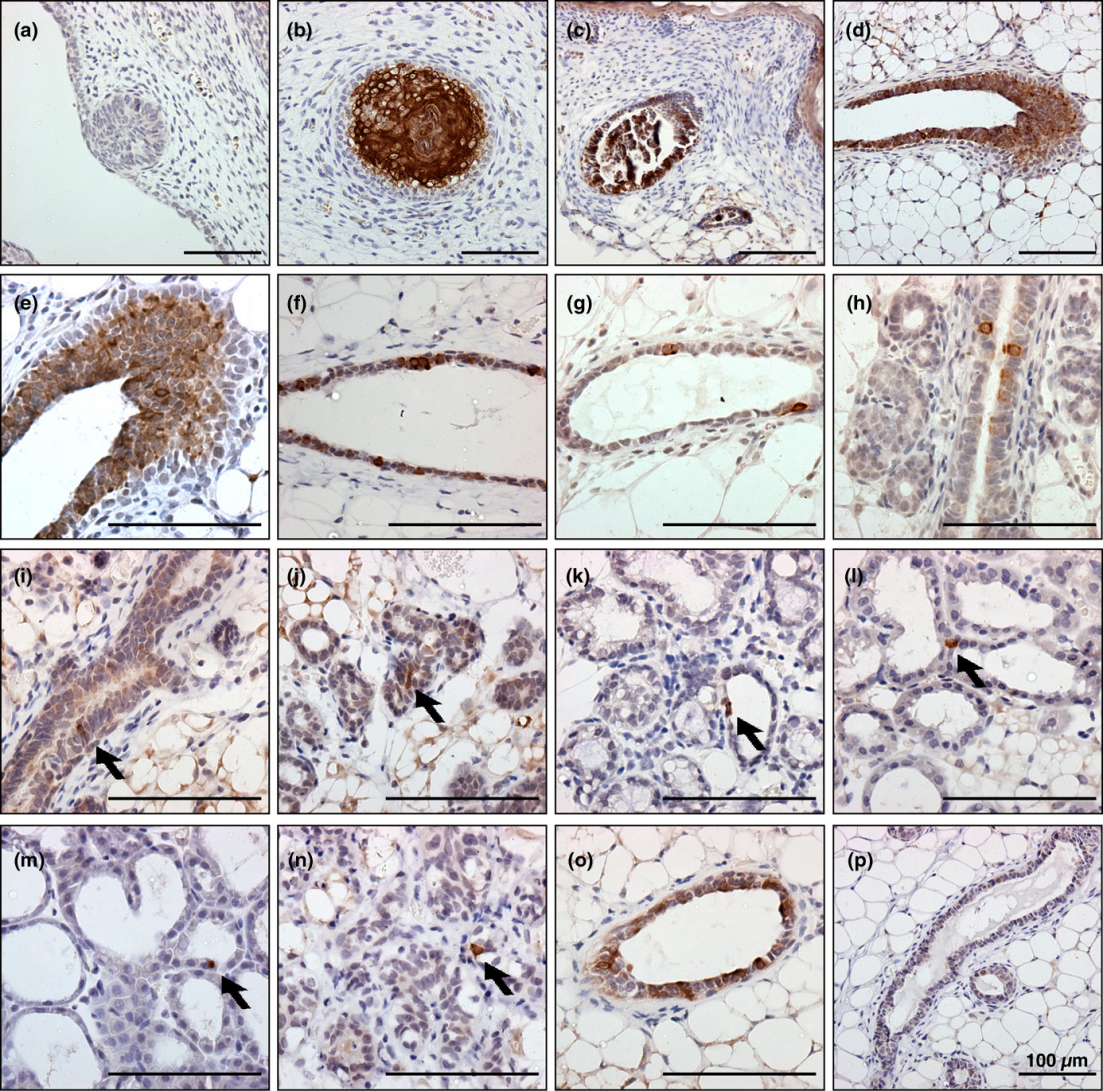
Keratin 6a (K6a) expression during mammary gland development. Tissues isolated from C57Bl/6 mice during various stages of mammary gland development were stained by immunohistochemistry with an antibody to detect K6a expression. **(a) **Embryonic day 14 mammary bud, **(b) **embryonic day 16.5 mammary bud, **(c) **day 5 pup, **(d,e) **6-week terminal end bud, **(f) **6-week pubertal duct, **(g) **12-week mature duct treated for 2 days with estrogen and progesterone, **(h) **mature duct after treatment for 21 days with estrogen and progesterone, **(i) **day 10 pregnancy, **(j) **day 15 pregnancy, **(k) **day 18 pregnancy, **(l) **lactation day 2, **(m) **lactation day 9, **(n) **involution day 3, **(o) **12-week mature duct from CCAAT-enhancer-binding protein-β-null mammary gland, **(p) **12-week mature duct from K6ab-null mammary gland. Scale bar, 100 μm.

K6 expression has been reported previously in the body cells of TEBs from pubertal mice [15,16], but its expression earlier in mammary gland development has not been reported. Paraffin-embedded tissue sections from various stages of development were stained by immunohistochemistry with the use of an anti-K6a antibody (Figure 2). At embryonic day 14, expression of K6a was not observed in the mammary bud (Figure 2a), although positive staining was detected in other regions of the embryo (data not shown). By E16.5 (Figure 2b), when the center of the mammary bud begins to keratinize in preparation for formation of the lumen and branching, K6a expression was observed in the center of the bud but not in the outermost ring of epithelial cells and not in the mammary mesenchyme. Five days after birth, K6a expression was seen in most of the luminal epithelial cells making up the rudimentary ductal tree (Figure 2c). By six weeks of age, when TEB structures are infiltrating the fat pad, many of the body cells in the TEBs were positive for K6a, whereas the highly proliferative cap cell layer at the tip of the TEB did not express K6a (Figure 2d,e). Notably, some of the body cells showed more intense staining than others. The luminal epithelial cells in ducts from 6-week pubertal mice strongly expressed K6a (Figure 2f), but expression was markedly downregulated by 12 weeks in a mature gland (Figure 2g). K6 expression is associated with activated keratinocytes in the skin, a state of hyperproliferation [13], but after chronic (21 days) hormone treatment with estrogen and progesterone (E + P) to induce proliferation in the mammary gland, K6a expression remained sporadic (Figure 2h). The same was true throughout pregnancy at days 10, 15, and 18 (Figure 2i–k), lactation at days 2 and 9 (Figure 2l,m), and at day 3 of involution (Figure 2n). Very few K6a-positive cells could be found in sections from these stages of development (indicated by arrows). Mammary glands from C/EBPβ-null and K6ab-null mice served as positive and negative controls for K6a staining, respectively (Figure 2o,p). These patterns of expression are consistent with what is expected of a progenitor cell marker.

**Figure 3:**
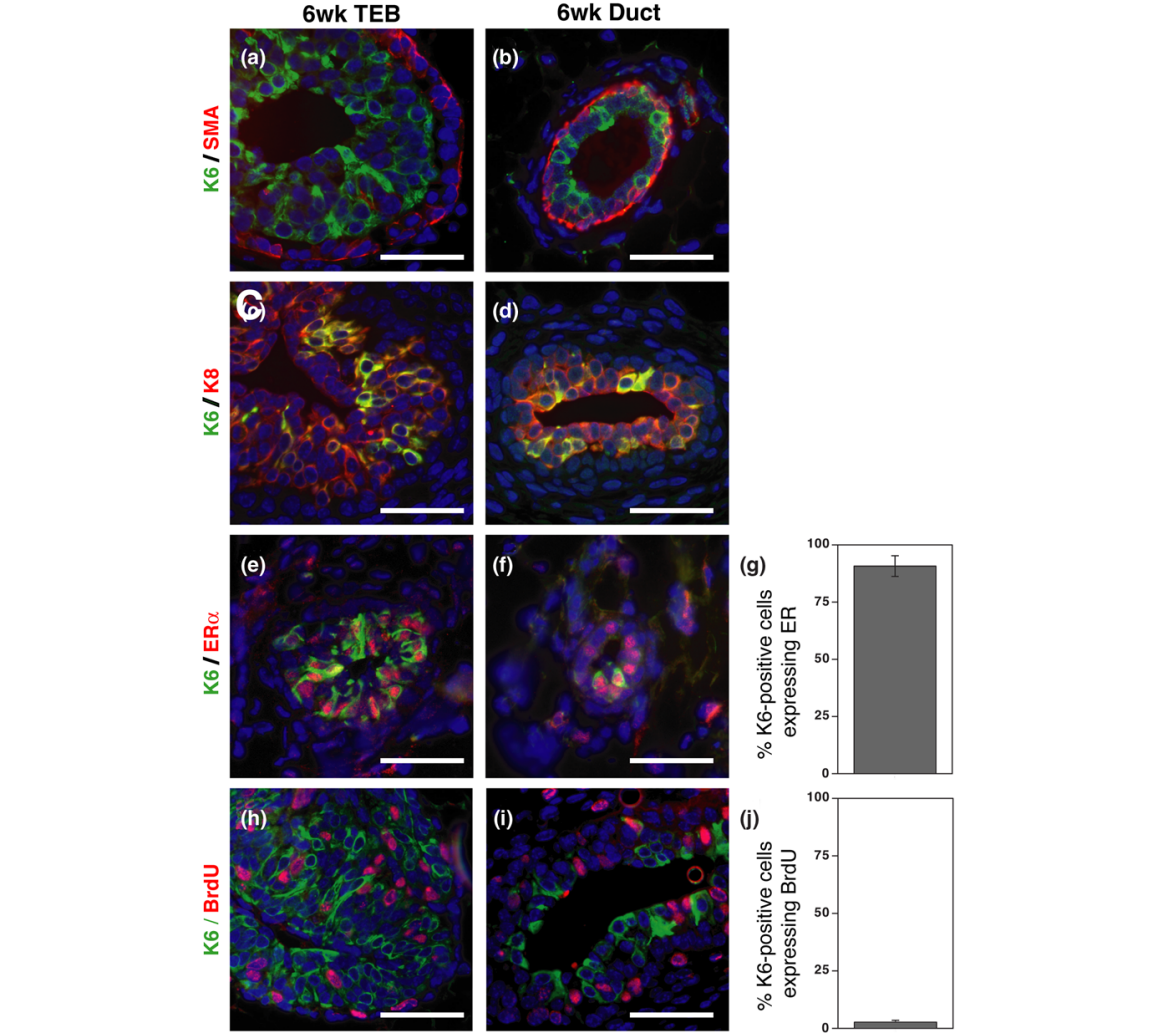
Co-localization of keratin 6a (K6a) expression with markers of mammary epithelium. Indirect immunofluorescence was performed on mammary gland sections from 6-week FVB mice, containing both terminal end buds (left column) and ducts (right column). K6a staining is shown in green for all sections. Co-localization was performed for smooth muscle α-actin **(a,b)**, K8 **(c,d)**, estrogen receptor α **(e,f)**, and bromodeoxyuridine **(h,i)**, which are all shown in red. Quantification of K6a-positive cells co-expressing estrogen receptor α or bromodeoxyuridine is shown in the bar graphs **(g,j)**. Scale bar, 50 μm.

To gain insight into the function of K6 during mammary gland development, double immunofluorescence staining was performed to co-localize K6a expression with known markers of mammary epithelium (Figure 3). Representative staining is shown from TEBs (left panels) or 6-week ducts (right panels). SMA was expressed in the cap cells of the TEB and in myoepithelial cells surrounding the ducts of the mammary gland (red; Figure 3a,b). However, K6a expression (green) was confined to the body cells of the TEBs and the luminal epithelial cells of ducts, and did not overlap with SMA expression (Figure 3a,b). K8 and ERα are markers of luminal MECs. Double staining for K6a with these proteins (Figure 3c–f) revealed nearly complete co-localization, with yellow fluorescence showing overlapping staining for K6a (green) with K8 (red; Figure 3c,d). More than 90% of the K6a-positive cells (green) expressed ERα (red; Figure 3e–g). Staining was also performed for K6a and BrdU, a marker of cells in S phase, to determine whether K6a was expressed in proliferating cells (Figure 3h,i). Only 2.6% of K6a-positive cells (green) incorporated BrdU (red). The K6a-positive cells that did co-localize with BrdU were those that expressed lower levels of K6a; the strongly K6a-positive cells were BrdU-negative.

**Figure 4:**
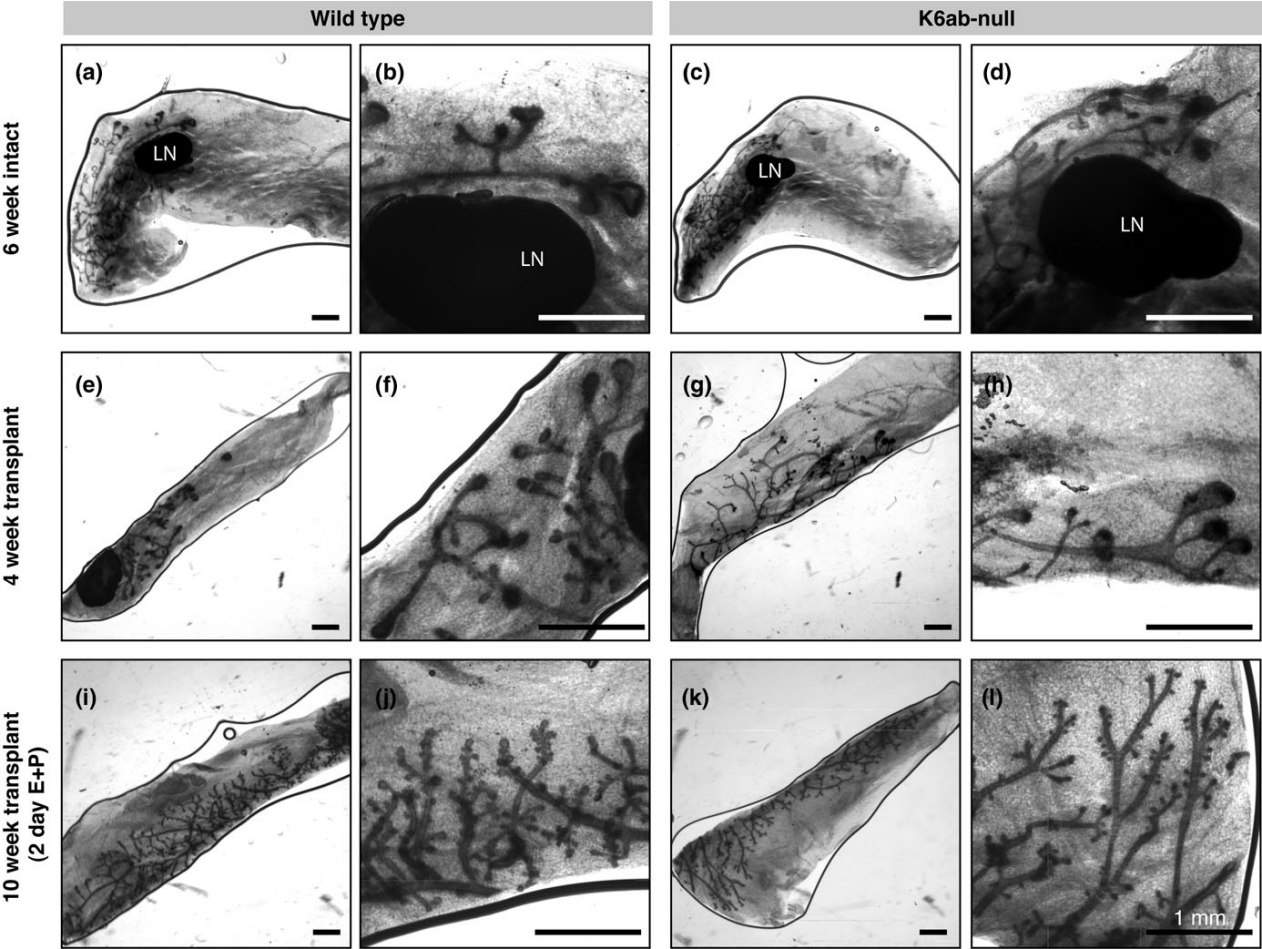
Keratin 6ab (K6ab)-null mammary gland whole-mount analysis. Mammary glands were isolated from intact wild-type (WT) or K6ab-null animals at 6 weeks **(a–d) **or transplanted WT or K6ab-null tissues were harvested after either 4 weeks of outgrowth **(e–h) **or 10 weeks of outgrowth after 2 days of treatment with estrogen and progesterone **(i–l)**. Whole-mount staining of the glands to reveal ductal structures is shown at two magnifications. Scale bar, 1 mm. LN, lymph node.

To determine a functional role for K6 in mammary gland development, the mammary glands from K6ab-null mice were analyzed. Double knockout mice, rather than K6a-null mice, were used to avoid potential compensation by K6b after deletion of K6a. Many K6ab-null mice die shortly after birth; a plaque of cellular debris on the back of the tongue, due to disintegration of the oral mucosa, prevents the pups from receiving proper nourishment [12,18]. However, about 25% of the null mice from the 129/SvEv background survive to adulthood [12]. Therefore, in addition to analyzing mammary glands from intact animals, tissues from WT and null mice were also transplanted and analyzed to rule out systemic effects on mammary gland development. Whole-mount staining of glands from intact 6-week animals (Figure 4a–d) revealed normal ductal development in WT and K6ab-null glands; the TEBs had reached the lymph node in both genotypes and the structures looked morphologically similar. WT and K6ab-null mammary tissues were transplanted into the cleared fat pads of host mice and allowed to grow for four weeks. Outgrowths from these transplants (Figure 4e–h) also displayed similar phenotypes and morphology, suggesting that neither K6a nor K6b is essential for ductal elongation in mammary gland development. Transplanted tissues were also allowed to grow out for ten weeks to fill the fat pad fully. Acute hormone treatment with E + P for two days was given to induce proliferation of mammary epithelial cells. Outgrowths from both WT and mutant tissues were indistinguishable, and both responded to the hormone treatment as shown by alveolar budding along the ducts (Figure 4i–l).

**Figure 5:**
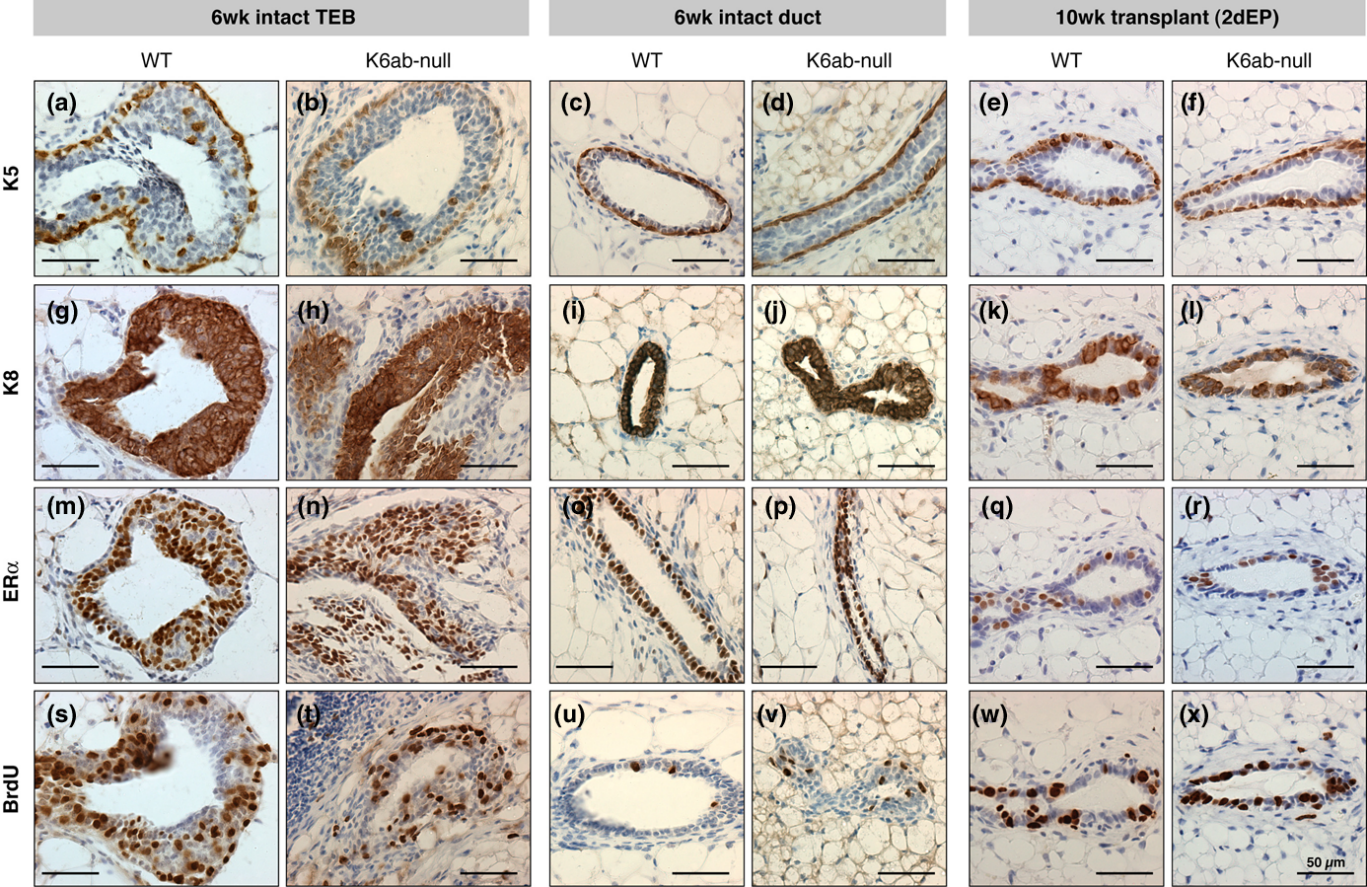
Marker expression in keratin 6ab (K6ab)-null mammary glands. Mammary gland sections from 6-week intact animals, including terminal end buds (left column) and ducts (middle column), or from transplanted tissue after 10 weeks of outgrowth and treatment for 2 days with estrogen and progesterone (right column), were stained by immunohistochemistry for markers of mammary epithelium, including K5 **(a–f)**, K8 **(g–l)**, estrogen receptor α **(m–r)**, and bromodeoxyuridine **(s–x)**. Scale bar, 50 μm.

Next, immunostaining for markers of mammary epithelium was performed on sections from the WT and K6ab-null glands to discern any molecular differences that might exist between the two genotypes. Representative images from 6-week intact glands, both TEBs and immature ducts, and from 10-week outgrowths treated for 2 days with E + P, are shown in Figure 5. K5 is a marker of myoepithelial cells, and positive staining is observed surrounding the TEBs, in the cap cell layer and myoepithelial cells, as well as a few cells that infiltrated into the body cells (Figure 5a,b). K5 staining showed a uniform pattern in myoepithelial cells surrounding the immature ducts (Figure 5c,d) and more punctate staining around mature ducts (Figure 5e,f). Antibodies against K8 and ERα stained most of the body cells in TEBs from WT and K6ab-null glands, but these proteins were not expressed in the cap cells (Figure 5g,h,m,n). Most of the luminal epithelial cells in ducts from 6-week animals uniformly expressed K8 and ERα (Figure 5i,j,o,p), but this expression was downregulated and became non-uniform in the mature gland (Figure 5k,l,q,r). Finally, immunostaining for BrdU incorporation was performed to detect cells in S phase. Both the body and cap cells in TEBs were highly proliferative (Figure 5s,t). Few BrdU-positive cells were detected in the 6-week ducts (Figure 5u,v), but higher levels of BrdU incorporation were observed in mature ducts after hormone treatment (Figure 5w,x). In all cases, the WT and K6ab-null samples showed similar staining patterns, suggesting that there were no obvious molecular changes in the K6ab-null glands.

**Figure 6:**
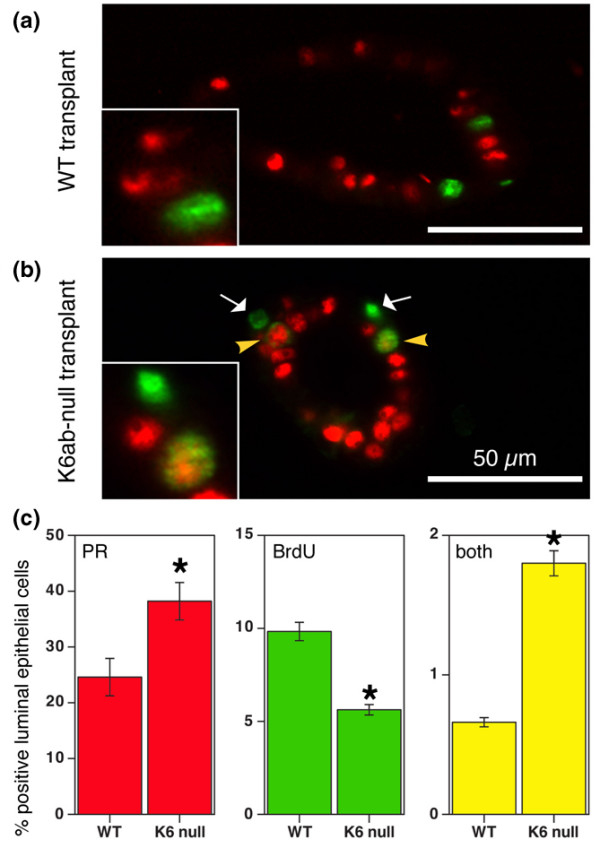
Progesterone receptor (PR)/bromodeoxyuridine expression and co-localization in keratin 6ab (K6ab)-null mammary glands. Mammary gland sections from wild-type **(a) **or K6ab-null **(b) **transplanted tissue after 10 weeks of outgrowth were treated for 2 days with estrogen and progesterone and stained for PR (red) and BrdUrd (green) by indirect immunofluorescence. The inset images show increased magnification of double-positive cells. White arrows indicate proliferating myoepithelial cells. Scale bar, 50 μm. **(c) **Cells positive for each marker, or those that co-localized (yellow arrowheads), were quantified and expressed as a percentage of total luminal epithelial cells. Error bars represent the SEM, and asterisks indicate statistical significance (*p *< 0.05).

One of the hallmarks of normal mammary gland development and patterning is the dissociation of steroid receptor expression from proliferating cells in the mature gland [19-21]. ERα and PR co-localize, but cells expressing steroid receptors are rarely BrdU-positive. Because most K6a-positive cells expressed steroid receptors and were not proliferating, we wanted to see what happened to PR and BrdU expression after loss of K6ab. WT and K6ab-null mammary tissues were transplanted and allowed to grow out for 10 weeks, then treated for 2 days with E + P to induce proliferation. By whole-mount analysis, it appeared that the K6ab-null transplants responded to hormone treatment as well as WT, indicated by the alveolar budding observed (Figure 4c,d). Indirect immunofluorescence was used to stain sections for both PR (red) and BrdU (green) and the percentages of MECs positive for PR, BrdU or both were quantified (Figure 6a–c). The K6ab-null transplants had a higher percentage of PR-positive MECs (*p *< 0.05), and lower levels of proliferation than WT transplants (*p *< 0.05). Whereas PR/BrdU co-localization was rare in WT glands (0.6%), there was a threefold higher occurrence of double-positive cells in K6ab-null glands (1.8%). Not only did a higher percentage of MECs co-express PR and BrdU, more of the fields counted contained the double-positive cells (22% for WT versus 56% for null). So, although loss of K6ab does not seem to significantly alter mammary gland development, including ductal elongation and response to pregnancy hormones, increased co-localization of steroid receptor expression and proliferation was observed.

## Discussion

This study shows that the expression of K6 is highest during the initial development of the gland and becomes sporadic in the mature gland. K6a expression was confined to cells destined to become the luminal epithelial cells of the mammary gland. Even as early as E16.5, K6a expression was detected in the innermost cells of the mammary bud, as opposed to the outer ring of epithelial cells that are p63-positive (SLG and JMR, unpublished observation). Similarly, the body cells of TEBs, as opposed to the cap cell layer, expressed K6a. K6 expression in the epidermis has previously been shown to be regulated by epidermal growth factor and tumor necrosis factor-α signaling pathways [22-24], but nothing is known about what regulates its expression in subpopulations of cells in the mammary anlage or TEBs.

Our original observation of an increased number of K6a-positive cells in the mature mammary glands from C/EBPβ-null mice correlated with a block in development, suggesting an accumulation of more primitive cells [6]. Several other groups have also observed K6-positive cells in their mouse models of mammary gland development, supporting the idea that K6 may indeed be a putative marker of progenitor cells in the gland. For example, Stingl and colleagues have recently described a population of mouse mammary cells called 'colony-forming cells' or Ma-CFCs that were isolated by fluorescence-activated cell sorting based on a CD24^high^CD49f^low ^profile and occurred with a frequency of about 1 in 63 cells [5]. Although these cells did not display outgrowth potential in fat pad transplantation experiments, Ma-CFCs were defined by Stingl and colleagues as progenitor cells that are able to grow discrete colonies on low-density adherent cultures. Both mRNA and protein levels of K6 were enriched in these Ma-CFC cells, which also had increased expression of other luminal cell markers, such as K8, K18, and K19.

Notch signaling has been implicated in stem cell self-renewal [25]. Recombination signal binding protein, J-type (RBP)-J is the common transcriptional mediator of Notch receptors. Targeted deletion of RBP-J in the mammary gland resulted in a transient increase in K6 expression in luminal epithelial cells during early pregnancy [26]. These results suggested an arrest at an immature stage of mammary gland development, similar to that in the C/EBPβ-null mice.

In addition to being a marker of early mammary gland development and putative progenitor cells, K6 expression has been observed in a subpopulation of cells in both mammary hyperplasias and tumors induced by transgenic expression of Wnt-1, β-catenin, or Myc [7]. However, hyperplasias and tumors induced by polyoma middle T antigen, Neu or H-Ras are more homogeneous and do not express K6. Li and colleagues therefore suggested that some tumors might arise from the amplification of progenitor cells, whereas the others might promote differentiation of the progenitors, thereby depleting the population.

Lisanti and colleagues have reported increased K6 expression in the hyperplastic mammary ducts of caveolin-1-null mice, along with increased β-catenin expression [27]. They proposed that activation of the Wnt/β-catenin pathway led to the accumulation of mammary progenitor cell accumulation. Finally, overexpression of the proto-oncogene Met, a receptor tyrosine kinase, under the control of the MSCV (mouse stem cell virus), resulted in non-progressive mammary neoplasms [28]. These lesions contained large numbers of K6-positive cells, again suggesting that this might represent the expansion of a progenitor cell population. However, when Met was overexpressed under the control of MMTV (mouse mammary tumor virus), no neoplasms were detected and K6 expression was not observed.

Keratinocytes activated by injury or stress express K6 and migrate to the site of wound healing [13]. Although K6 expression has been associated with hyperproliferation, expression of K6 in the epidermis does not overlap with the incorporation of [^3^H]thymidine [29], supporting our observation that K6-positive cells are mostly quiescent. Instead, it is possible that K6 regulates a migratory function required in progenitor cells so as to permit their dispersal throughout the mammary gland. However, deletion of K6a and K6b did not seem to have any detectable effect on ductal elongation in mammary glands from intact animals or in outgrowths from tissue transplants.

The lack of an overt mammary gland phenotype in the K6a/b double knockout mice was not unexpected, because it has been difficult to demonstrate the functional properties of most markers used to isolate and characterize stem and progenitor cells by means of gene deletion in genetically engineered mice. For example, deletion of one of the best-characterized mammary stem/progenitor cell markers, CD49f (α6-integrin), did not have any reported mammary gland developmental phenotypes in transplants of the null mammary anlage [30]. This unexpected result might be considered surprising, because integrins have been suggested to have an essential function in adhesion in the stem cell niche [31]. Furthermore, conditional deletion of CD29 (β1-integrin) resulted in impaired alveolar development and lactation [32], with no reported effect on ductal morphogenesis [32]. Although the triple knockout mouse for Brcp1 (Abcg2), Mdr1a, and Mdr1b resulted in the loss of side population (SP) cells, a phenotype defined by the ability of these cells to efflux Hoechst 33342 dye associated with stem/progenitor cells, there was no mention of a mammary gland development phenotype in these mice [33].

A key experiment to test the functional effect of loss of progenitor cell markers requires limiting dilution serial transplantation to distinguish long-term versus short-term engraftment of stem/progenitor cells deleted for specific genes. This has only recently been accomplished for CD49f and CD29 [4]. It is most likely that many of these markers may be only just markers, and may not have critical functions. However, notwithstanding this caveat, it is crucial to identify lineage markers as tools for the characterization of different mammary cell types. K6 seems to be one such useful lineage marker.

In the present study, an increase in the number of proliferating PR-positive cells was observed after the loss of K6a/b. K6a-positive cells co-localized with steroid receptor expression, but rarely with BrdU incorporation, a marker of proliferation. Normally, steroid receptor expression does not overlap with proliferation [19-21]. However, inappropriate co-localization of steroid receptors with proliferative markers is often found in pre-neoplastic disease [34-36]. A recent study suggests that activated TGF-β acts in an autocrine manner to prevent ERα-positive mammary epithelial cells from proliferating [37]. In addition to an increased number of K6a-positive MECs, C/EBPβ-null mice have increased levels of activated TGF-β as well as increased expression of PR and decreased proliferation in the mature mammary gland, further supporting this hypothesis [6,38]. Interestingly, ERα and PR have also been proposed to be markers of mammary progenitor cells, representing a quiescent population scattered throughout the gland hypothesized to self-renew through asymmetric division [39,40]. Human mammary epithelial cells sorted for expression of p21 or Msi-1, suggested to be putative stem cell markers, also had enriched expression of steroid receptors. Additionally, SP cells had sixfold enrichment of ERα-positive cells in this model system; however, K6 expression was not analyzed [40]. Alternatively, recent studies with a xenograft model of T47D human breast cancer cells expressing ER plus different PR isoforms demonstrated increased K6 mRNA expression in tumors in response to treatment with E + P that was dependent on the expression of PR [41]. Thus, the precise relationship between steroid receptor and K6 expression in normal mammary epithelial cells, and how this might be altered in breast cancer, remains to be established, but it is interesting to speculate that K6 might have a function in this interaction.

## Conclusion

Although K6a may not be essential for mammary gland development, the loss of both K6a and K6b resulted in an increased number of PR-positive MECs and decreased proliferation after exposure to steroid hormones. There was also increased co-localization of PR and BrdU, suggesting alterations in patterning events important for lobuloalveolar development. Although K6 does not seem to have a function in progenitor cells, it may still provide a useful lineage marker for studies of mammary gland development and tumorigenesis.

### Note added in proof

Interestingly, an unexpected function for keratin 17, a potential keratin 6 partner, in regulating protein synthesis and epithelial cell growth has been reported recently [42].

## Abbreviations

BrdU = bromodeoxyuridine; C/EBP = CCAAT-enhancer binding protein; E = embryonic day; E + P = estrogen and progesterone; ERα = estrogen receptor α; K5 = keratin 5; K6 = keratin 6; K8 = keratin 8; MEC = mammary epithelial cell; PR = progesterone receptor; SMA = smooth muscle α-actin; SP = side population; TEB = terminal end bud; WT = wild-type.

## Competing interests

The authors declare that they have no competing interests.

## Authors' contributions

SLG contributed to the conception and design of the study, carried out tissue transplants, whole mount staining, immunostaining experiments, quantitation and statistics, interpretation of data, and figure preparation, and drafted the manuscript. WB performed immunostaining experiments, fluorescent image capture and quantification. MAL, DRR, and YL participated in the design and coordination of the study and reviewed the manuscript. JMR contributed to the conception and design of the study and helped prepare the manuscript. All authors read and approved the final manuscript.
